# Supplementary material for: Cluster randomized controlled trial to assess the effectiveness of a package of community-based interventions on continuum of maternal and newborn healthcare in Sidama, Ethiopia: The SiMaNeH trial protocol
Source: PLoS One. 2025 Apr 17;20(4):e0310341. doi: 10.1371/journal.pone.0310341 (PMC12005492; doi:10.1371/journal.pone.0310341)
Supplement: S1 File — (DOCX) [file pone.0310341.s001.docx]

Cluster randomized controlled trial to assess the effectiveness of a package of community-based intervention on continuum of maternal and newborn healthcare in Sidama, Ethiopia: **The SiMaNeH-Trial Protocol**

**Developed by**:

Achamyelesh Gebretsadik (MPH, PhD, Associate Professor, Hawassa University- project leader)

Yaliso Yaya (Mphil, PhD, Associate Professor, Western Norway University of Applied Sciences)

Yemisrach Shiferaw (MPH, PhD candidate, Hawassa University)

Hirut Gemeda (MSc, PhD candidate, Hawassa University)

June 2023

Hawassa, Ethiopia

**Collaborating institutions:**

School of Public Health, Hawassa University, Ethiopia

Sidama Regional State Health Bureau, Ethiopia

Centre for International Health, University of Bergen, Norway

Western Norway University of Applied sciences, Norway

**Funding**

This project is part of NORHED-II (SENUPH-II) funded projects in collaboration between Hawassa University, Ethiopia and University of Bergen, Norway

Contents

[Summary iii](#_Toc116721955)

[Acknowledgement v](#_Toc116721956)

[Acronyms and abbreviation vi](#_Toc116721957)

[Introduction 1](#_Toc116721958)

[Statement of the problem 3](#_Toc116721959)

[Significance of the study 7](#_Toc116721960)

[Literature review 8](#_Toc116721961)

[Community based care 8](#_Toc116721962)

[Roll of community health workers 8](#_Toc116721963)

[Community based neonatal health care and its packages 12](#_Toc116721964)

[Trends in prevalence of Neonatal deaths 12](#_Toc116721965)

[Causes and Timing of Neonatal Death 13](#_Toc116721966)

[Overall goal 14](#_Toc116721967)

[Objectives for the group leader 14](#_Toc116721968)

[Methods and materials 16](#_Toc116721969)

[Study area 16](#_Toc116721970)

[Study period 17](#_Toc116721971)

[Overview of study designs 17](#_Toc116721972)

[Source and study population 18](#_Toc116721973)

[Inclusion and exclusion criteria 18](#_Toc116721974)

[Description of intervention(s) and main exposures 18](#_Toc116721975)

[Outcome measures 22](#_Toc116721976)

[Sample size 23](#_Toc116721977)

[Randomization and allocation 23](#_Toc116721978)

[Sampling procedure 24](#_Toc116721979)

[Data collection 24](#_Toc116721980)

[Data quality control 25](#_Toc116721981)

[Data entry, analyses and processing 25](#_Toc116721982)

[Outcome measures definitions 25](#_Toc116721983)

[Ethical considerations 27](#_Toc116721984)

[Dissemination of study results 27](#_Toc116721985)

[Flowchart of the study 28](#_Toc116721986)

[Tentative time schedule 32](#_Toc116721987)

[Reference 33](#_Toc116721988)

[Annex I: 38](#_Toc116721989)

#

# Summary

**Background:** **Background:** Maternal and newborn mortality and morbidity remain high in low- and middle-income countries such as Ethiopia. Limited access and dropouts from essential continuum of care interventions are critical factors. In Ethiopia about one in five complete the continuum of essential care through pregnancy, childbirth, and postnatal period Ethiopia. Evidence is limited on whether a package of interventions involving key community health actors increase the proportion completing essential maternal and newborn healthcare continuum in rural Sidama regional state, Ethiopia.

**Objective:** This study aims to implement and evaluate the effectiveness of community-based interventions designed to enhance involvement of key community health actors to improve completion rate of continuum of maternal care and utilization of newborn care.

**Methods:** Twenty rural kebeles (clusters) in Sidama Regional State, Ethiopia, are randomly allocated to intervention and control arms. A total of 2000 pregnant women, 1000 per arm, will be recruited between 20^th^ and 26^th^ week of gestation after intervention. Then they will be followed until six weeks postpartum between June 2024 and February 2025. In the intervention arm, mothers and newborns will receive targeted interventions at home and in their community through a package of interventions designed to improve completion rate of recommended maternal and newborn care. Control clusters will receive normal care from the state public health system. Primary outcomes will be the difference in the completion of continuum of maternal care and essential and emergency newborn care between intervention and control clusters measured by composite indicator constructed from variables. Secondary outcomes include rates of antenatal care completion, facility deliveries with skilled care, completion of at least four postnatal care, essential newborn care, and emergency identification and referrals, mortality measures.

**Conclusion:** This trial will implement and evaluate community-based intervention package within existing community healthcare infrastructure to produce evidence for informed policy and practice to achieve improved community-based healthcare.

**Budget:** A tentative budget plan will be **2,569,415**birrs, which will be secured from NORHED-II (SENUPH-II) project.

Key words: continuum of care, maternal, neonatal health care, community resource group.

# Acknowledgement

First, we would like to express our gratitude to Hawassa University's College of Medicine and Health Sciences, School of Public Health, for establishing and supporting this maternal and neonatal health research core group on a project that allows us to work as a research team. Second, we would like to express our heartfelt gratitude to professor Bernt Lindtjørn for his mentorship through the process of developing this proposal and prior application for funding. We also want to express our gratitude to Dr Taye Gari, coordinator of NORED II (SENUPH-II) project at Hawassa University for the trust and support to this core research team. We appreciate Norwegian Program for Capacity Development in Higher Education and Research for Development (NORHED) for financing for the project through NORHED-II (SENUPH-II) funding program.

# Acronyms and abbreviation

ANC Antenatal Care

BCG Bacille Calmette- Guérin

BEmONC Basic Emergency Maternal and Neonatal Obstetric Care

CHW Community Health Worker

DE Design Effect

ECD Early Childhood Development

EDHS Ethiopian Demographic and Health Survey

EPI Expanded program of Immunization

FMOH Federal Ministry of Health

HC Health Center

HDSS Health Demographic Survey Sites

HDT Health Development Team

HEP Health Extension Program

HEWs Health Extension Workers

HIV Human Immuno-deficiency Virus

ICC Intra Cluster Correlation

ICMNCI Integrated community based Management of Newborn and Childhood illness

IFHP Integrated Family Health Program

IRB Institutional Review Board

IUCD Intra Uterine Contraceptive Device

LBW Low Birth Weight

MEDHS Mini Ethiopian Demographic Health Survey

NMR Neonatal Mortality Rate

PMTCT Prevention of Mother to Child Transmission

PNC Postnatal Care

PHCU Primary Health Care Units

PSBI Possible Serious Bacterial Infection

SBA Skilled Birth Attendant

SDG Sustainable Development Goal

SENUPH South Ethiopia Network of Universities in Public Health

SPH School of Public Health

STI Sexually Transmitted Illness

SRS Sidama Regional State

TBAs Traditional Birth Attendants

UNICEF United Nations International Children`s Emergency Fund

WDG Women Development Group,

WHO World Health Organization

**Introduction**

Global maternal and child mortality was remarkably reduced between 2000 and 2015 through efforts under Millennium Development Goals (MDGs). However, recent estimates by UN inter-agency highlight that the progress stagnated between 2016 and 2020 (1). In 2020, there were over 300,000 maternal deaths, 2.4 neonatal deaths, and another 1.9 stillbirths (2, 3). Furthermore, neonatal mortality, the death of a newborn baby within the first month of life, shares nearly half (47%) of mortality of under-5 children shows slow progress.

Over 99 percent of maternal and newborn deaths and severe morbidities and disabilities from pregnancy and childbirth occur in low-income and middle-income countries. Unfortunately, sub-Saharan Africa has the highest neonatal mortality rate in the world with 27 per 1000 live births (4), also contributing close to half (42%) of global stillbirths, the death of babies after 28^th^ weeks of gestation and before birth, in 2019 (5). Poor investments (6) and limited access to quality maternal and newborn care in resource-limited settings are important factors because one-third of the neonatal deaths occur within the first day and three-forth in the first week after birth (3). Estimates from modeling 75 high-burdened countries highlighted improved coverage of essential maternal and newborn care interventions could avert over two-third of neonatal deaths, one-third of stillbirths, and over half of maternal deaths every year (7) because 85% of newborn deaths are because of fetal asphyxia, preterm birth, intra-partum complications and neonatal infections (7).

Since the WHO’s Alma Ata declaration in 1978 (8), community-based interventions, intended to bring essential healthcare services closer to these hard-to-reach areas have emerged as important alternatives to improve maternal and newborn healthcare. However, the strategies and its success are not uniform across communities and countries.

Ethiopia, like other low-income countries, struggles with limited access to the life-saving interventions for maternal and newborn health to the larger part of its population living in the rural areas, underscoring the need for innovative and sustainable strategies to close the gap in adverse pregnancy and childbirth outcomes.

One such innovative approach is the Ethiopian Health Extension Program (HEP), a flagship national program rolled out in 2003 by Ministry of Health of Ethiopia (9). In each village of about 5,000 population, two women known as Health Extension Workers (HEWs) trained for one year in general health are placed in a satellite health post to provide health promotion and disease prevention services to residents. These women are permanent employee of Ethiopian government with monthly salary.

The Health Extension Program (HEP) has been instrumental in expanding access to primary healthcare services, demonstrating the feasibility and impact of deploying health extension workers (HEWs) in rural settings. From 2012, another voluntary network of about 30 women per village known as women development team (WDT) were introduced to support the works of HEWs, creating a strong network closer to households. Consequently, a systematic review by Yibeltal Assefa and colleagues showed that the HEP has significantly improved essential preventive and health promotion indicators HEP (10). Yet, concerning the skills of the HEWs to provide essential maternal and newborn care, a study identified up to 88% of HEWs had poor knowledge of neonatal danger signs and expressed that they are not confident enough to attend deliveries (11). This underscores the importance of an approach that improves their confidence, skills, and integration with health facilities.

Furthermore, a 2022 article by Tiruneh GT and colleagues demonstrated that overall, only one-fifth (20%) of women who initiated the first antenatal care completed the continuum of recommended essential care that combines completion of antenatal care, skilled birth attendance, and critical postpartum care (12).

One of the challenges in maternal and newborn care continuum in poorly organized health systems is low proportion of women having postnatal contact with competent health professionals. In 2022, WHO published guidelines for postnatal care and recommends at least four such contacts in the key time periods during the first 24 hours, 48-72 hours, one-two weeks and six weeks after birth (13). Without successful completion of postnatal care during these key periods, it is difficult to succeed in improving the continuum of care.

The low rate of completion of essential continuum of care highlights the importance of a community-based maternal and newborn care system that supports the mother, the baby and family through key community health stakeholders such as skilled and motivated community health workers, trained and convinced community opinion leaders and women support groups. A systematic review synthesized evidence from community-based interventions to improve maternal and newborn health by Zohra S Lassi and colleagues in 2015 showed that interventions with strong community involvement showed significant positive results compared to other community-based intervention without community involvement (14). Previous community-based intervention trials in low-resource settings have demonstrated the important benefits of community-based interventions (15-17). However, many of these trials have been limited by a narrow focus of addressing specific factors, overlooking the complex and interconnected nature of the various community-based actors and stakeholders that influence healthcare utilization in rural communities.

Considering these challenges, this protocol outlines a comprehensive approach to conducting cluster randomized rural trials implementing a package of interventions that includes enhancing involvement of several community actors. The current intervention includes training and supervision and evaluate the effectiveness of these interventions in improving the completion of maternal continuum of care and newborn essential care.

Our hypothesis is that the proposed community-based intervention package intended to engage a broad spectrum of key actors, including community health workers, women's support groups, influential community leaders, local social welfare scheme systems (known as IDIR in Ethiopia), and the linkages between community and health facility services has a potential to improve maternal and newborn healthcare continuum care outcomes compared to control group receiving the routine public health services. Continuum of maternal and newborn care is a continuity of care during pregnancy, childbirth, and postpartum period with coordinated efforts from home through the health facility (18). To achieve improved rate of completion of the continuum of care, we aim to address the complex barriers such as poor knowledge of the importance of initiation and continuation of care until completion of standard care, transport barriers, and family care related challenges when a mother or baby needs to travel to reach care.

## Statement of the problem

Every year, more than 60 million women give birth at home without the benefit of skilled care. Each year, half million women die as a result of pregnancy and childbirth complications. The majority of these mortalities occur in sub-Saharan Africa and South Asia [[19](#_ENREF_19)].

Ethiopia have reduced maternal mortality rate from 720 in 2000 to 412/100,000 live birth in 2020. These are great achievement , but still too high[[20](#_ENREF_20)]. The global agenda is through sustainable development goals (SDGs) is to reduce maternal mortality ratio to less than 70 per 100,000 live births and this requires substantially improved maternal health service [[21](#_ENREF_21)].

Maternal causes of death are classified in to direct and indirect cause of death. Three fourth of maternal death are due to the direct cause of maternal mortality among this. Keeping the regional variation in place globally among the direct cause of maternal death hemorrhage , pregnancy induced hypertensions and sepsis were the leading cause [[22](#_ENREF_22)]. In Ethiopia three major direct causes resulted in three fourth of maternal death, these are hemorrhage, hypertensive disorders, puerperal sepsis[[23](#_ENREF_23)]. Receiving all package of skilled care during antenatal care (ANC), delivery and postnatal period by all women and children at the appropriate level and time reduce mortality and improved health status. Women in rural area deprived of access; as a result there is disparity in utilization of maternal services between urban and rural[[24](#_ENREF_24), [25](#_ENREF_25)]. Furthermore, low level of taking the recommended ANC on time and low completion of the continuum of care (ANC4+, SBA, and PNC within 2 days after birth) by rural women was very indicating inaccessibility for skilled attendant[[26](#_ENREF_26), [27](#_ENREF_27)].

According to a study done in Ethiopia. Only 5.1% of mothers in Ethiopia were referred by health extension workers, and 40% of those mothers ended up in hospitals for routine ANC, family planning, and child vaccinations[[28](#_ENREF_28)]. Even though health extension workers are expected to spent half of their time in community to perform home visiting to make continuum of maternal care to rural health care un accessed mothers by doing home based postnatal care[[15](#_ENREF_15)] there role is unsatisfactory in home visiting and postnatal care and the coverage is very low [[16](#_ENREF_16), [17](#_ENREF_17)]and also many of the content of postnatal care are missing and only few received during the critical time[[18](#_ENREF_18)]. The current health extension optimization guideline also encourage home based postnatal care, since culturally if normal delivery appeared most women will not get out of home until 40 days[[13](#_ENREF_13), [14](#_ENREF_14)]. One of the intervention plans is to enhance home-based postnatal care in the community.

Effective referral system enhances the utilization of primary health care and ensure for client in receiving cost effective and timely service at all level based on the case they have. Referral initiated when the condition of the patient is beyond the capacity of the facility and patient is also interested to do so and bidirectional with feedback and back referral to lower level too [[18](#_ENREF_18), [29](#_ENREF_29)].

A qualitative study conducted in northern part of Ethiopia identified a problems in the referral linkage, in which a referred mothers face poorly prepared reception at health center when they are referred from community and inappropriate facilitation of referral from health center to hospital and also lack of formal feedback to the health post for referred cases[[30](#_ENREF_30)].

Worldwide, 2.4 million newborns died in the first 28 days of life in 2019. There are approximately 7 000 newborn deaths every day, especially in sub-Saharan Africa and South Asia ([26](https://mail.google.com/mail/u/0/#_ENREF_26)). A systematic review conducted in Kenya and South Africa showed that in 2018, the Neonatal Mortality Rate (NMR) for Kenya was 19.6 deaths per 1000 live births and the NMR of South Africa was 10.7 deaths per 1000 live births ([27](https://mail.google.com/mail/u/0/#_ENREF_27)). 50% neonatal mortality occurs in the first 24 hours after birth and 75% of neonatal mortalities occur within the first week of life of the newborn ([28](https://mail.google.com/mail/u/0/#_ENREF_28)). In Ethiopia, about 87,000 neonates die annually, of which 85% of births are at home ([28](https://mail.google.com/mail/u/0/#_ENREF_28)). A prospective cohort study conducted in the Aroresa district in the Sidama region showed that the neonatal death was 41 per 1000 live births during the study period ([29](https://mail.google.com/mail/u/0/#_ENREF_29)). The majority is preventable through simple, low-cost interventions and one-half or more of these deaths occur in the home ([30](https://mail.google.com/mail/u/0/#_ENREF_30)).

The main causes of death are asphyxia, hypothermia, hypoglycemia, infection, and complications related to prematurity[[31](#_ENREF_31), [32](#_ENREF_32)]. A prospective cohort study conducted among neonates in Northern Ethiopia in 20 showed that two thirds of deaths were attributable to prematurity 23 (34%) or asphyxia 21 (31%). The other study showed that prematurity takes the part in the early neonatal period (37%), while asphyxia (35%) was more common in the late neonatal period[[33](#_ENREF_33)]. Another study showed the association that, preterm babies were 2.2 times higher risk than term developing birth asphyxia. Similarly, the low weight of the neonate had also a significant association with birth asphyxia[[34](#_ENREF_34)].

The other causes of deaths are related to receiving low quality of care associated with inadequate contact throughout the continuum of care[[35](#_ENREF_35)]. According to WHO, quality of care is defined as *“health care which is safe, effective, timely, efficient, and equitable and people centered*”[[36](#_ENREF_36)].If the community health workers are not providing all elements of essential neonatal care and not visiting the neonate in all of the contact time recommended by the WHO, it is considered as low quality care given by HEWs[[37](#_ENREF_37), [38](#_ENREF_38)].In addition to this, delays related to the caretaker in problem recognition or in deciding to seek care in the community accounts for 50% of neonatal deaths[[32](#_ENREF_32)].

Community health workers are well situated geographically and socially to create a bridge between providers of health, and community services that may have difficulty in accessing these services and reduce the prevalence of death [[39](#_ENREF_39), [40](#_ENREF_40)]. Although they are recognized as a central and critical resource for achieving national and the health-related Sustainable Development Goals of Universal Health Coverage and reducing neonatal mortality in many low-income settings[[9](#_ENREF_9), [10](#_ENREF_10)], the knowledge, and skills of the community health workers towards sick newborn care are still low, they did not identify cases and refer them to health facilities[[11](#_ENREF_11), [12](#_ENREF_12)].

Quality of neonatal health care practice is determined by the knowledge level of community health workers[[41](#_ENREF_41)]. In this regard, most community health workers have inadequate knowledge. A study conducted in Nigeria to assess the retention of knowledge and skills of primary health care workers towards basic newborn resuscitation to manage asphyxia in newborn revealed that out of 106 birth attendants, no participant had adequate knowledge and only one had adequate skills at baseline [[42](#_ENREF_42)]. A similar study conducted in Nigeria to explore the perception and experience of neonatal resuscitation among community health workers showed that the majority (88.2%) had poor knowledge of neonatal resuscitation. The overall knowledge of community health workers about neonatal resuscitation was poor as compared to expectations[[43](#_ENREF_43)].

The adequacy of the knowledge of community health workers can be associated with the adequacy of basic or refresher training they received[[44](#_ENREF_44)]. They are health care suppliers who live within the community they serve and get lower levels offormal instruction and preparing than proficient health care laborers such as medical attendants and specialists[[45](#_ENREF_45)]. A trial done in South Africa revealed that the postnatal care performance of CHWs before receiving health educational intervention or training was poor. The effect of not receiving additional training by CHWs is also evidenced by scoring low knowledge level of postnatal mothers towards the care given to her in that community (only 30.3%) [[46](#_ENREF_46)]. A qualitative study conducted in rural Nigeria, some key informants proposed that “ *there should be concerted efforts to train and retrain the existing cadres of community health workers via the effective implementation of the task shifting policy in Nigeria, in addition to possibly revising the existing training curricula, instead of introducing community midwifery*” [[47](#_ENREF_47)].

An implementation and scale-up lessons from eastern Uganda showed that the effect of training improved the knowledge of CHW`s from 41.3% to 77.4% and their performance [[48](#_ENREF_48)]. A systematic review showed that provision of continuous training for CHWs was associated with rise gratification and inspiration. This leads to increase their confidence in the ability to conduct their responsibilities [[49](#_ENREF_49)]

So far, different studies have been shown the gap of health workers who are working at health centers, hospitals and health posts towards neonatal health care. Interventions also implemented only to improve the gap of health workers working at health centers and hospital level. But, there is limited evidence of action taken to improve the gap of community health workers who are working in the community or at home in the place where 85% of maternal and neonatal mortality occurred [[50](#_ENREF_50)]. Therefore, the main purpose of this study is to improve the quality of community based maternal and neonatal health care through enhancement of community health workers` performance at Sidama Regional state.

#

# Significance of the study

Our intervention will focus on improving the quality of maternal and newborn health care services provision through maximizing the knowledge and skill of the HEWs and Community Health Development Teams (CHDTs).This will be realized through skill training and mentorship on BEmONC. Therefore, the aim of this proposal is to assess the baseline knowledge and skill of the health extension workers towards maternal, neonatal and child health care service and intervene the identified gap so, it will improve the quality of maternal and newborn care services at grass root level. It will also improve the performance of the HEWs and HDTs and their job satisfaction by filling gaps of their knowledge and skill on basic maternal and child services. Enhance community based maternal and child health service uptake. In turn, this will have paramount importance in reduction of maternal neonatal and child morbidity and mortality.

It also provides evidence for policy makers, FMOH, regional bureau, zonal health department and for all health workers for further improvement and revision of the service implementation strategy.

# Literature review

**Health care system in Ethiopia**

Ethiopian health care system brought significant change following the first in its kind of the health policy development since 1993 after 5o years[[51](#_ENREF_51)]. Before that the health system give emphases for the curative and urban centered, delivered with few health facilities and health care professionals, with small government expenditure which is far from the World Bank estimated for sub-Saharan African country.

## Community based care

Community based care can be described in different health care situations: health facilities, community settings through different specialties like maternal and newborn care. The care is often provided by CHWs and may include home visitations and other intervention packages. According to Lassi study, community-based care is a vital constituent for delivery of a continuum of care in low – resource communities like Ethiopia [[52](#_ENREF_52)].

As different studies indicated that the concept of community-based maternal and newborn care is more relevant in relation to community-based care interventions, and these services are important for reducing neonatal mortality rates and hence the program is viewed as good to improve neonatal survival[[53](#_ENREF_53)]. In Ethiopia, although the community based maternal and neonatal health care is given by skilled birth attendants (SBAs) who are accredited health professionals such as midwives, doctors and nurses at health facility level, the system has resulted in key challenges regarding the accessibility and availability of SBAs[[53](#_ENREF_53), [54](#_ENREF_54)]

## Roll of community health workers

The roll of community health workers is broadly used to deliver care for a broad range of health issues [[55](#_ENREF_55)]. Research findings in India and Bangladesh had revealed that community health workers could diagnose and treat neonatal infections when referral was not possible, resulting in reduced mortality[[56](#_ENREF_56), [57](#_ENREF_57)]. Nepal had been an early leader in scaling newborn care to communities and provided health education and counseling, support for outreach services, distribution of health commodities, and provision of sick-child care[[58-60](#_ENREF_58)]

**Health extension workers and maternal health care utilization**

A qualitative study revealed that, HEWs have community trust since they are from the community they are working. However, due to lack of confidence and skill, the health extension workers call TBA and manage together delivery case in the community. There is high demand from community to give birth in health post for these HEWs were promised after receiving sufficient training and get necessary material to provide services for delivering mother[[30](#_ENREF_30)].

Frequent visit of the health extension enhanced the utilization of health service during pregnancy. Among the specific services HIV testing was less utilized before health extension worker practice has shown significant increment especially who have been counseled at household level than not. This indicates the role of health extension program in increasing service utilization of the community especially in maternal health [[61](#_ENREF_61)].

Implementation of package-based intervention was done to reduce maternal mortality by strengthening the health care delivery through upgrading the existing health system in Gamo Gofa zone in southern Ethiopia. The intervention consists of training non clinician physician, provision of material health care, birth and death registry and training of health extension workers for safe and clean delivery. The study has shown significant maternal mortality reduction and improvement in maternal service Uptake. This study has contributed for policy direction in Ethiopia except it lacks comparison and difficulty of controlling for confounder due to the nature of the study [[62](#_ENREF_62)].

Interventional study to increase community participation for maternal health increased the utilization of pregnant women forum and health development team lead meetings in southern Ethiopia. And also from before after intervention result increased percentage of pregnancy identified referred by HDA, ANC and skilled delivery utilization in the community [[63](#_ENREF_63)].

In order to evaluate the effectiveness of the implementation strength of basic emergency obstetric and newborn care (BEmONC) at primary health care level in rural communities, a before-and-after evaluation study was conducted in four large regions of Ethiopia (Oromiya, Amhara, Southern, and Tigray) in 91 districts and 134 health centers. The results revealed a dose-effect relationship in which health facilities having high BEmONC implementation score have increased institutional delivery and high infant survival rates. Additionally, it was found that even acknowledged trained professionals (health officials, midwives, and nurses) who had taken BEmNOC for procedures requiring a certain level of expertise, such as infant resuscitation and manual placenta removal, did not significantly differ from the baseline study.

In western Kenya a cluster randomized control trial study was conducted among pregnant women who presented to health facilities for their first antenatal care visits by 32 weeks gestation to improve maternal and newborn care quality in the community. Group based community health volunteer led health education were provided for 60-90 minutes two times a month a total of (3hrs)/month every 15 days for the study period and control group receive monthly home visiting and standard health education by community health volunteers between 27 November 2017 and 8 March 2018 to see primarily increased institutional delivery outcome. Improvement was seen in institutional delivery, postnatal care within 48 hours post-delivery, exclusive breast feeding, family planning usage and completion of immunization. Whereas no difference in antenatal care uptake between the control group and intervention group[[64](#_ENREF_64)].

**Maternal health service**

Skilled maternal health care utilization (skilled antenatal care, delivery and postnatal care) was low in mothers in rural Ethiopia far from targeted plan at national level or globally. Inappropriate perception about the utilization of ANC assuming that when pregnant mother has health problem will receive the ANC make them not to utilize ANC. While nearly one third of mothers were inaccessible either due to road or delayed ambulance unable to utilize institutional delivery [[65](#_ENREF_65)]. Even if maternal health care provided for free of payment for the service including ambulance service [[66](#_ENREF_66)] in Ethiopia, the utilization of institutional delivery is not satisfactory by rural mothers, since distance from home to health facility and having poor awareness on birth preparedness hinder the utilization[[67](#_ENREF_67)].

HEWs community service facilitated as a teamwork with women development groups (WDG), in which they serve as a source of information for early identification of pregnant mother in a community and further management and referral linkage when need arises. A qualitative study showed that, effectiveness of the HEWs in life threatening maternal condition will determine further trust[[68](#_ENREF_68)]. There is over all increment of service usage in continuum of cares during pregnancy after the implementation of HEP [[69](#_ENREF_69)], the improvement of maternal and child health knowledge and health seeking behavior has significantly linked with model households[[70](#_ENREF_70)].

Majority (50-74%) of Ethiopian women 50% - 74% of give birth deliver at home[[20](#_ENREF_20), [71](#_ENREF_71)] skilled attendant and well informed people round mothers are critical to save life for early identification of complication and referral To reach the unreached population in the rural the health extension workers was perfect in increasing the access. The skill of this huge work force needs to be improved in continuum of care during pregnancy and child birth in identifying managing and referring mothers. The rural population, which makes up the majority of the population, has had difficulty accessing health services. Improving access to emergency obstetric care requires expanding services that are publicly available, such as district hospitals and health centers. While obstetric surgery (such as cesarean birth for obstructed labor) necessitates the use of a hospital, many other life-saving operations can be performed in health centers and first-aid stations. Treatment and first assistance (e.g., manual removal of the placenta and injection of ergometrine) for postpartum hemorrhage, which can kill in a matter of hours, must be provided at the most remote level of the health care system[[72](#_ENREF_72)]. Participatory learning with women's groups can improve the effectiveness of care reduce maternal and newborn mortality by a large amount clinical officers in action and antenatal care uptake from skilled health providers and health extension workers whereas delivery care has not shown a change from skilled provider and TBAs brought better utilization than HEWs [[73](#_ENREF_73)].

**Antenatal care**

Provision of effective antenatal care prevents significant number of maternal mortality, science a quarter of maternal death due to pre-eclampsia /eclampsia and hemorrhage occurs before birth(Antepartum hemorrhage), during pregnancy and can be preventable[[23](#_ENREF_23)].

Study conducted in rural Kenya to assess the quality of ANC care in two ways one in service provision by asking the women by list of recommended services and the other asking women experience of care mostly to know the information sharing and communication following each care provision. The result showed that there is sub optimal care in both cases while most mothers received the basic health cares like measuring blood pressure and urine test at least once during ANC follow up[[74](#_ENREF_74)].

Longitudinal observational study reviled that low quality of ANC received by pregnant women in health facilities found in Hossana town in southern Ethiopia. The quality of care was measured among skilled health care professionals was nearly half mothers did not received physical examination and only one third received history taking whereas laboratory test and counseling were below that [[75](#_ENREF_75)].

**Post-natal care**

Study identified that, low utilization of maternal service in study area (ANC 45% labor and delivery 40.7 % and it was very low for postnatal care from the national coverage which was 14.3%. it was suggested that by health professionals who participated in clarifying the reason in the service utilization study, low postnatal utilization might be due to inability of the health facilities to keep mothers after delivery for 24 hours[[76](#_ENREF_76)]. In Ethiopia, only 14.5 % of mothers revived postnatal care within first 24 hours by health extension workers[[20](#_ENREF_20)] it is believed that, since it is common cultural practice to maintain postnatal mothers until 49 (40)days at home to get res[[13](#_ENREF_13)], The new optimization of health extension program implementation guideline recommends to provide postnatal care at home setup[[77](#_ENREF_77)] .

## Community based neonatal health care and its packages

Community based neonatal health care is part and parcel of community based care that has grown into a popular idea in neonatal health care worldwide [[53](#_ENREF_53), [78](#_ENREF_78)]. Community – based neonatal health care is a way of delivering life-saving care to mothers and neonates at the community level within the Ethiopian health system [[79](#_ENREF_79)] to achieve its main goal that is reducing neonatal mortality through the provision of high quality maternal and neonatal health services and community demand creation[[8](#_ENREF_8)]. In this package, there are nine components are included. These are: early identification of pregnancy, provision of focused antenatal care, safe and clean delivery, promotion of health facility delivery, provision of immediate newborn care, including application of chlorohexidine on cord, recognition of asphyxia, initial stimulation and resuscitation of newborn baby, prevention and management of hypothermia, management of pre-term and/or low birth weight neonates, management of neonatal sepsis/very severe disease at community level[[6](#_ENREF_6), [8](#_ENREF_8), [50](#_ENREF_50), [80](#_ENREF_80)].

## Trends in prevalence of Neonatal deaths

Worldwide, 2.4 million newborns died in the first 28 days of life in 2019. There are approximately 7 000 newborn deaths every day, especially in sub-Saharan Africa and South Asia[[81](#_ENREF_81)]. A systematic review conducted in Kenya and South Africa showed that in 2018, the Neonatal Mortality Rate (NMR) for Kenya was 19.6 deaths per 1000 live births and the NMR of South Africa was 10.7 deaths per 1000 live births[[82](#_ENREF_82)]. About 50% and 75% of neonatal mortalities occur in the first 24 hours after birth and within the first week of life of the newborn, respectively[[31](#_ENREF_31)]. In Ethiopia, about 87,000 neonates die annually, of which 85% of births are at home [[50](#_ENREF_50)]. A recent prospective cohort study conducted in Aroresa district in Sidama region showed that the neonatal death was 41 per 1000 live births in the study period[[83](#_ENREF_83)]. The majority are preventable through simple, low-cost interventions and one-half or more of these deaths occur in the home [[84](#_ENREF_84)]

## Causes and Timing of Neonatal Death

The main causes of death are asphyxia, hypothermia, hypoglycemia, infection, and complications related to prematurity[[31](#_ENREF_31), [32](#_ENREF_32)]. A prospective cohort study conducted in 2017 among neonates in Northern Ethiopia in showed that two thirds of deaths were attributable to prematurity 23 (34%) or asphyxia 21 (31%). The other study showed that prematurity takes the part in the early neonatal period (37%) , while asphyxia (35%) was more common in the late neonatal period [[33](#_ENREF_33)]. Another study showed the association that, preterm babies were 2.2 times higher risk than term developing birth asphyxia [AOR = 2.20; 95% CI (1.02, 4.76)]. Similarly, the weight of the neonate had also a significant association with birth asphyxia[[34](#_ENREF_34)]. A study conducted in eastern Uganda showed that major (50%) contributing delays to newborn death were caretaker delay in problem recognition or in deciding to seek care were 50%[[32](#_ENREF_32)].

So far, different studies have been shown the gap of health workers who are working at health centers, hospitals and health posts towards neonatal health care. Interventions also implemented on health workers at health centers and hospital level. However, there is limited evidence of actions to improve the quality of services and capacity of health extension workers. Therefore, the main purpose of this study is to improve the quality of community based maternal and neonatal health care through enhancement improving of health extension workers performance at Sidama Regional state.

**Outcome variable**

Reproductive health history factors

Parity, Gravidity , complication

Gestational age at first ANC, Receiving ANC 4+ number of children,

Reproductive health utilization related factors

Distance from health, Age at first birth, distance from the health facility, women autonomy, and mass media exposure, lack of information, inadequate and poor quality services, cultural beliefs,

**Socio demographic economic factor**

Age, religion, marital status, educational status of woman and spouse, occupation, Wealth index, residence

**Intervention**

Community support groups participatory learning action monthly meeting, Facilitation training for HEWs and for supporting health facility staff

ANC4+ utilization

Continuum of care

**Exposure variable**

Barrier and challenge of implementation of the intervention

Figure. Conceptual framework for continuum of care for maternal health adopted from different literature.

Output /Out come

**Process**

**Input**

Community based neonatal Health Care Intervention Packages

Postnatal Care

- Immediate and thorough drying
- Initiation of breathing
- Immediate skin-to-skin contact
- Delayed cord clamping
- Initiation of breastfeeding in the first hour
- Thermal care practice
- Identification, management & referral for neonatal Complications/danger signs

Improved neonatal health care practice (including the thermal care

**1.** Provision of training for health extension workers and health development team leaders

**2**. Participatory learning and action meeting with community health actors (CHWs, CHDTLs, Edir, religious, youth, kebele leaders, male agricultural dev. Leaders, and influential elderly peoples)

3. Regularmeetingfor discussion with HEWs+ HDTLs + community leaders (every month)

Increased rate of early initiated and exclusive BF

Improved early detection &Mgt of neonatal illnesses

3. Collaborative meeting

Improved referral system

Identification & referral with neonatal complications/danger signs

Fig.1. Conceptual framework of model for community based neonatal health care intervention adapted from International Initiative for Impact Evaluation & reading different literatures. Source: International initiative for impact evaluation: A systematic review 005

**Overall aim:**

The aim of the proposed trial is to evaluate the collective effectiveness of a package of community-based interventions on improving continuum of maternal and newborn healthcare.

**Objectives:**

1. To evaluate whether a set of community-based intervention improve completion of maternal continuum of care : A) The overall completion rate with composite result of combination of at least four antenatal care contacts, delivery in a health facility with skilled provider, and at least four postnatal care contacts and completion rates of each three components of the continuum of maternal care. B) Explore the experience of mothers and care providers on the effect of the intervention for maternal care, including the challenges and opportunities.
2. To determine the effectiveness of the community-based strategies on improving utilization of essential and emergency newborn care: A) WHO recommended four elements of essential newborn (immediate and thorough baby drying, skin-to-skin contact, initiation of early breast feeding within first hour, and delayed cord clamping). B) identification and referral of severely sick babies such as babies with danger signs. C) Cost-effectiveness of the intervention on improving maternal and newborn continuum of health care.

# Research question

1. What is the base line information of maternal continuum of care before the SeMaNiH trial intervention among intervention and control groups?
2. What will be the effect of SeMaNiH trial on ANC utilization among the intervention and control groups?
3. What is the effect of SeMaNiH trial intervention on the maternal continuum of care among intervention and control groups?
4. What are the barriers and challenges of the implementation of SeMaNiH trial intervention in the community?

# General objective for PhD candidate one

The general objective of this cluster randomized trial is to implement and assess the effect of package of community-based interventions on improving maternal health services between September 2023 and September 2024 in Sidama Region State, Ethiopia,

**Specific objectives**

1. To describe the pre-trial baseline status of drop out in the maternal continuum of care in terms of antenatal care utilization (ANC4+), Institutional delivery, postnatal care within 48 hrs. (Paper I)
2. To identify community based maternal health care practice received from community health workers among pregnant mothers in sidama regional state from March 2022 to March 2023 (Paper II)
3. To evaluate the effectiveness of package of community-based interventions on improving the proportion of institutional delivery and postnatal care with skilled health professionals( paper III)
4. To explore the challenges and opportunities related to capacitating the community health actors to improve maternal continuum of care (IV)

**PhD candidate 2 : Newborn (neonatal) Health**

# General Objective:

To determine whether a community-based capacity-building intervention aimed to improve the continuum of maternal and newborn care in Sidama regional state improves neonatal health care services utilization.

## Specific objectives:

1. To assess the base-line level health care service utilization of neonates who lived in selected study clusters in Sidama region in 2023 (base-line survey).
2. To determine whether a community-based capacity-building intervention (SiMaNeH trial) improves neonatal thermal care (measuring immediate skin-to-skin, thorough drying, head covering, and delayed bath) (Paper-II)
3. To asses the effect of a community-based capacity-building intervention (SiMaNeH trial) in improving timely initiation and exclusive breast-feeding rate during neonatal period in intervention group compared to control group (Paper-III).
4. To determine the effect of a community-based capacity-building intervention (SiMaNeH trial) in improving identification of sick neonates, and referral in the intervention group compared to controls (Paper IV).
5. To describe the cost-effectiveness of community-based capacity-building intervention to improve neonatal health care service utilization (Paper- V)

# Methods and materials

**Methods**

**Setting**

This trial research project will take place in rural areas of Sidama, a regional state in southern part of Ethiopia. Sidama became an autonomous regional state in 2020, separating from the former Southern Nations, Nationalities, and Peoples Regional State (SNNPRS) which is divided into four new regional states between 2020 and 2023. Sidama is situated approximately 400 kilometers south of Addis Ababa with an estimated population of about 4.6 million people in 2023. The capital of the regional state is Hawassa. Still, more than 80 percent of the population lives in rural areas, where agriculture is the primary economic activity and access to quality healthcare services is limited.

In 2018, an empirical study in one of the districts in Sidama showed that fertility was lower than reported from national estimates with crude birth rate of 22.8 births per 1000 population and total fertility rate of 2.9 children per woman. Furthermore, the population is transitioning to a low-mortality and low-fertility rate (19). However, the United Nations projected crude birth rate for Ethiopia is 30.6 births per 1000 population in 2024 (20), far higher than the empirical findings.


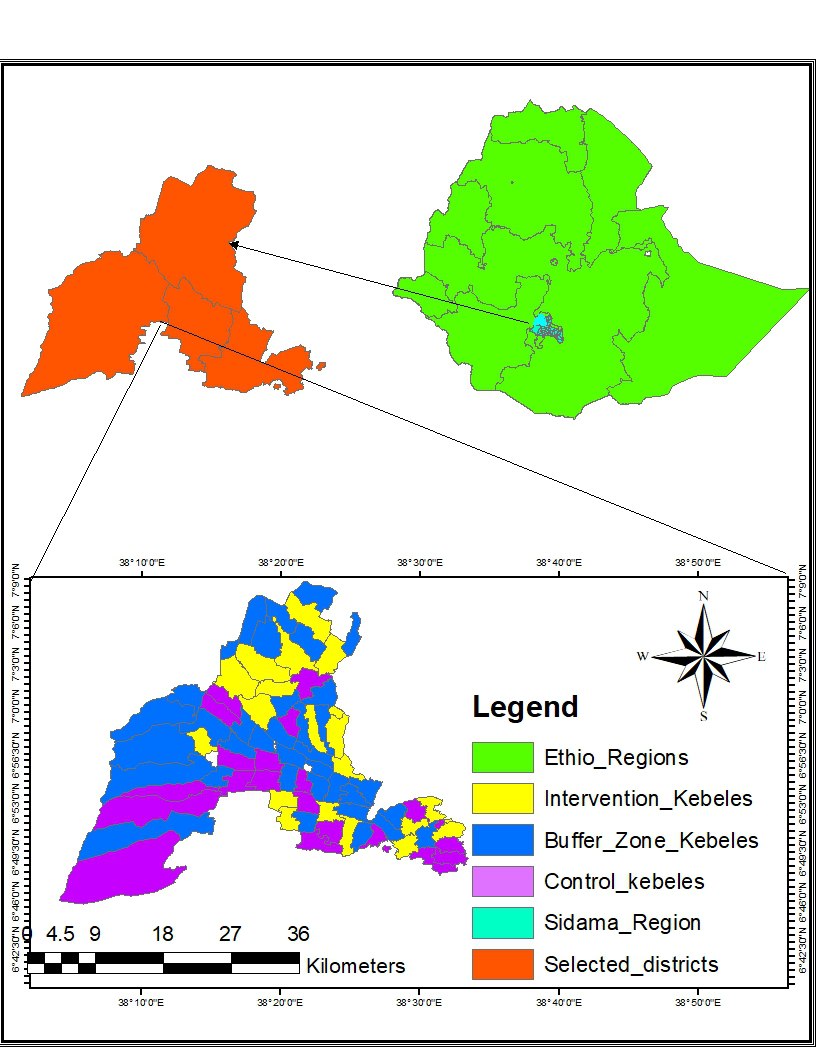


Figure 1: The map of the study area within Sidama regional State, Ethiopia.

Table 1: Background demographic and health service characteristics of study population

| Background | Intervention | Control | Remarks |
| --- | --- | --- | --- |
| Total population in 2024 | 84 347 | 87 830 |  |
| Expected births in 2024^x^ | 2 530 | 2 634 |  |
| Number of existing health centers | 7 | 8 | some overlaps |
| Number of Health Extension Workers | 37 | 35 |  |
| Number of Health Developments Team | 545 | 560 |  |
| Average distance to health centers | 5.3 km | 5,1 km |  |
| *Note: ^X^ = annual birth rate (3% of population) multiplied by population* | | | |

**Theoretical and conceptual frameworks:**

The trial will employ theoretical and conceptual frameworks, including the WHO-endorsed Participatory Learning and Action (PLA) model (21). The PLA will be used as a guiding model to be used by a team of community resource persons comprised of religious, administrative, and local social scheme leaders, and women support groups. The resource (support) group will use the model to identify, prioritize, address and re-plan based on previous achievements and weakness related to maternal and newborn care in their villages (22). In addition, the intervention will benefit from the process-based approaches of Theory of Change (TOC) principles (23). TOC model helps to understand and implement how activities, outcomes and impacts are connected to each other. Furthermore, WHO and Ethiopian Ministry of health principles of essential maternal and newborn care will be implemented (24, 25) to improve the continuum of care.

**Trial registration**

This trial is registered at Pan African Clinical Trial Registry and its number is PACTR202402782261294 and the link is <https://pactr.samrc.ac.za/TrialDisplay.aspx?TrialID=25464>

**Time Frame:**

| Table 2: Work plan for capacitating community health actors and other community groups in maternal continuum of care study |
| --- |

| **S/N** | **Activities** | **2021/2022 Academic Year** | | | | | | **2022/2023** | | | | **2023/ 2024** | | | | **2024/ 2025** | | | |
| --- | --- | --- | --- | --- | --- | --- | --- | --- | --- | --- | --- | --- | --- | --- | --- | --- | --- | --- | --- |
|  |  | **Sep- Nov** | **Dec - Feb** | **Mar -May** | | **Jun-Aug** | **Sep- Nov** | | **Dec - Feb** | **Ma -May** | **Jun-Aug** | **Sep- Nov** | **Dec - Feb** | **Ma -May** | **Jun-Aug** | **Sep- Nov** | **Dec - Feb** | **Ma -May** | **Jun-Aug** |
| 1. 1 | Developing final proposal |  |  | |  |  |  | |  |  |  |  |  |  |  |  |  |  |  |
| 1. 2 | Ethical issue secure |  |  |  | |  |  | |  |  |  |  |  |  |  |  |  |  |  |
| 1. 3 | Pilot Test tools |  |  |  | |  |  | |  |  |  |  |  |  |  |  |  |  |  |
| 1. 4 | Data collectors training |  |  |  | |  |  | |  |  |  |  |  |  |  |  |  |  |  |
| 1. 5 | Implementation of the Intervention |  |  |  | |  |  | |  |  |  |  |  |  |  |  |  |  |  |
| 1. 6 | Follow up |  |  |  | |  |  | |  |  |  |  |  |  |  |  |  |  |  |
| 1. 7 | Collecting data |  |  |  | |  |  | |  |  |  |  |  |  |  |  |  |  |  |
| 1. 8 | Data analysis |  |  |  | |  |  | |  |  |  |  |  |  |  |  |  |  |  |
| 1. 9 | Writing First draft report |  |  |  | |  |  | |  |  |  |  |  |  |  |  |  |  |  |
| 1. 10 | Writing Final Report |  |  |  | |  |  | |  |  |  |  |  |  |  |  |  |  |  |
| 1. 11 | Attending conference internationally |  |  |  | |  |  | |  |  |  |  |  |  |  |  |  |  |  |
| 1. 12 | Presentation of findings |  |  |  | |  |  | |  |  |  |  |  |  |  |  |  |  |  |
| 1. 13 | Submitting the final report |  |  |  | |  |  | |  |  |  |  |  |  |  |  |  |  |  |
| 1. 14 | Manuscript writing and sending for journals |  |  |  | |  |  | |  |  |  |  |  |  |  |  |  |  |  |
| 1. 15 | Final dissertation report |  |  |  | |  |  | |  |  |  |  |  |  |  |  |  |  |  |

**Ethical approval**

The study was approved by the institutional review board (IRB) of Hawassa University in Ethiopia (IRB/363/15) issued on August 07/2023. Permission for the study was received from Sidama Regional State Health Bureau and subsequent local authorities. All participants will be asked to give a written consent information sheet to be presented to them and attached with this protocol. Participants will be informed of the aim of the study and their freedom to refuse or participate and withdraw from the trial freely.

**Study design**

This study will use a cluster randomized controlled trial with two arms of equal size. We use villages (kebeles in Ethiopian structure) with an average population of about 5,000 people as the clusters served as units of randomization.

**Randomization technique and Blinding**

We selected 20 clusters randomly from eligible 80 from four districts in the region and allocated them into intervention and control in 1:1 allocation,10 each to the intervention and control cluster. To balance based on the distance of clusters from nearby health facilities between intervention and control clusters, we used stratified first the 20 clusters into four categories based on the population size and distance to the nearest health facility to reduce confounding. The four strata were: 1) Higher population (over 4,000 residents) and longer distance (more than an hour of walking distance), 2) Higher population and shorter distance (less than an hour walking distance 3) Lower population (less 4,000 residents) and shorter distance and 4) Lower population and longer distance. Then clusters from each stratum were randomly allocated to intervention and control using a computer-generated random number using simple randomization. A seed number was produced and sent from a university of Bergen as a starting point for random number generation. Blinding is difficult because of the nature of cluster randomized trials, but we created a buffer zone between intervention and control clusters to reduce intervention contamination. Data collectors are independent of the intervention process and do not have formal information on which study arm the cluster to which they collect data belongs.

**Sampling strategy and sample size**

To calculate the sample size of the participating mothers and babies in the study, we used the sample size calculation for cluster randomized controlled trials with the fixed number of clusters recommended by Karla Hemming and colleagues (26). We fixed the number of clusters to 20 for logistic and access reasons. We base our estimate of pregnant women with annual crude birth rate of 30 births per 1000 population per United Nations Population Division projection for Ethiopia in 2024 (20). The average pregnant mother-baby pair per cluster was estimated to be 150 during the study period. To estimate the minimum required sample, the trial aims to increase the completion rate of the continuum of pregnancy, birth, and postpartum care from an estimated 20% exiting status to 30% in intervention clusters with a 95% significance level and 80% power. We take the intra-cluster correlation of 0.01. Substituting these numbers in the following formula gives the minimum number of mother-baby pairs for the study. We used the following formula to calculate the sample size.

$$n=\frac{\left( Z\alpha/2+Z\beta\right)^{2}\times\left( p1\left( 1-p1 \right)+p2\left( 1-p2 \right) \right)}{\left( p1-p2 \right)^{2}} \times\left( 1+m-1 \right) \times ICC$$

- *n* is the sample size = number of pregnant mother-baby pairs needed per study arm
- *Z_α/2_* is the z-value corresponding to the significance level (α=0.05, Zα/2 =1.96).
- *Z_β_* is the z-value corresponding to the desired power (β = 0.20, Zβ = 0.84).
- *p_1_* is the expected proportion in the control group (0.20), the continuum of care completion in control clusters.
- *p_2_* is the expected proportion in the intervention group (0.30), expected continuum of care completion in intervention clusters.
- *m* is the average cluster size = 150 mothers per cluster on average.
- *ICC* is the intra-cluster correlation coefficient (0.01).

This provides a minimum of 723 pregnant women-baby pairs per trial arm, resulting in a total sample size of 1446. However, because several outcome measures may need more samples and to have adequate numbers for sub-analysis, we decided to recruit a total of 2000 woman-baby pairs, 1000 in each arm.

**Participants:**

**Inclusion criteria:**

We shall include mothers who permanently reside in the study clusters and consent to participate. All pregnant mothers who fulfill the above criteria and do not meet the following exclusion criteria will participate. This includes mothers in their gestational period of between the 20th and 26th weeks within the four months of the recruitment period (June-September 2024).

**Exclusion criteria:** We will not recruit mothers with less 20th weeks of gestation because of longer time needed for follow-up. In addition, mothers whose pregnancy terminated before 20th week of gestation will not be included because the main emphasis of the study is on completion of maternal care and utilization of newborn care. Mothers who do not consent to participate in the study after information will not be included.

**Intervention**

To achieve the aim of improving the completion rate of the continuum of essential care from pregnancy care through postnatal care, the intervention package uses four mechanisms of action to help reduce barriers to essential healthcare utilization. The four mechanisms of action are: 1) Actively identify and connect to healthcare services, 2) Educate and prepare at home, 2) Support emotionally and materially through local collective efforts, and 4) Provide home-based essential care in case when care from health facilities is not actual.

To implement the four mechanisms of action stated above, the project coordinates the efforts of three existing community health stakeholder groups through training and supervision. The three stakeholders are the community health workers (HEWs in Ethiopia), the women support groups (HDTs in Ethiopia), and the community resources groups, also termed community opinion leaders (COLs).

HEWs are women permanently employed on average two in all clusters (Kebeles) in Ethiopia and paid monthly salary from the government. They have overall responsibility in their clusters for health promotion, disease prevention, and provision of basic maternal and newborn care such as antenatal, postnatal, and baby resuscitation. HEWs will participate majorly in three of the four mechanisms of action, namely identification, educating and preparing, and provision of home-based essential care when delivery happens at home, in addition to routine postnatal care. Furthermore, they will coordinate the work of COLs for the local collective support efforts.

WDTs are about 30 voluntary groups of women in each cluster residing in sub-villages of clusters in Ethiopia, freely helping the works of HEWs. They have recognition from the government without getting paid. Because of their unique position closer to sub-villages, they have the unique opportunity to identify mothers as they become pregnant in their respective villages and connect to healthcare services. They also educate and prepare mothers and families for standard and emergency care. WDTs' main contribution to the intervention is actively identifying and connecting mothers and babies to essential standard care and emergency care when they observe danger signs. WDTs will give particular attention to high-risk and hard-to-reach mothers and newborn babies such as extremely poor, living in difficult topography and distant villages from the health facilities and HEWs stations as well as those with existing sicknesses and malnutrition. As such, the contribution of the village WDTs in the four mechanisms of action will be identifying and connecting, education and preparing and involving with COLs in strengthening financial, transport, and family care support through collective local efforts so that a mother or baby reaches to health facility and receives care.

Community opinion leaders (COLs) are key leaders who lead opinions such as approval of the importance of receiving healthcare services and coordinating local collective help mechanisms. COLs are a group of local leaders forming a committee which includes influential religious leaders, cultural leaders, local government administrative leaders, and leaders of local welfare scheme called IDIR in Ethiopia. IDIR is a local welfare system where families contribute financially or materially every month. This welfare system often shares serious costs during deaths and severe sicknesses.

The main responsibility of the COLs is identifying problem such as barriers hindering mothers and babies receiving important health care and fall out of the continuum of care. They respond to the problem coordinating local collective efforts, for example transport or financial support to reach a health facility and taking care of the family members at home. For this contribution, they meet every two-weeks to evaluate and re-plan their actions based on the principles of Participatory Learning and Action (PLA) model (21). WDTs help COLs with gathering and providing sub-village information. COLs use their high level of acceptance to visit and support families where the relationship between the woman and her husband is not smooth and put great pressure on the health and wellbeing of women or their newborns. At such households, COLs provide essential advice on the importance of care for a pregnant women and newborn babies. This is expected to contribute to a mother and baby receive essential care that improve their survival and wellbeing.

The project provides training and supervision support to the three key stakeholders participating in intervention.

For COLs, the training and supervision will be based on PLA model of problem identification, providing local solutions, evaluating the effect, and re-planning process. COLs receive a day-long initial training and a two-monthly supervision discussion for an hour during the project period.

Trainings and supervision to HEWs will have two dimensions: On one hand, HEWs will be trained and supported on technical refresher issues related to baby resuscitation and home-based immediate support when a birth occurs at home, and essential postnatal maternal and newborn care and on the other hand, they will be trained on leadership role to lead the efforts of COLs and WDTs. HEWs receive two days of intensive training followed by two-monthly supervision and monitoring meetings with the project team.

The training and supervision of WDTs will be mainly on identifying and connecting mothers and babies for normal essential care as well as detecting danger signs and referring for emergency care babies and mothers with severe sickness (danger signs). In addition, WDTs receive training on maternal essential self-care, preparation for healthcare, nutrition, rest, hygiene, and other important information which in turn they will teach mothers and families. Further, the WDTs receive training on tracking and giving special attention to high-risk mothers and babies in difficult families and hard-to-reach villages so that they are not left out or fall out of the critically needed healthcare services. Through these mechanisms, the project builds a supportive network around mothers to help improve the initiation and completion of essential services in the continuum of care around pregnancy and childbirth. Table 2 below describes the roles of each stakeholder to the four mechanisms of actions to improve continuum of care in the intervention clusters.

Table 2: The role of stakeholders in the community-based actions in intervention clusters

|  | 1. Identify and connect | 1. Educate and prepare | 1. Support through collective efforts | 1. Home-based essential care |
| --- | --- | --- | --- | --- |
| HEWs | All pregnant mothers for essential ANC, delivery, and postnatal care in collaboration with HDTs and COLs  Mothers and babies in high-risk of falling out of care, or even do not initiate first contact.  Screen and recruit mothers for study 20-26^th^ week of gestation. | Mothers and families on essential care, nutrition, hygiene…  Prepare mothers and family for facility delivery, emergency health seeking. | Mainly through coordinating the efforts of COLs | Encourage antenatal care as per the guidelines of MoH  Emergency maternal and newborn help for deliveries occurred at home.  Routine postnatal care for mothers and babies. |
| HDTs | Identify mothers and babies in her sub-village and connect to HEWs, COLs and health care.  Guide data collectors to locate homes. | Educate and prepare mothers and families on essential care, nutrition, hygiene, emergency care | Inform to COL s and HEWs about mothers and babies not receiving care | Educate and prepare.  Do not provide any technical care. |
| COLs | Identify mothers and babies at high-risk and hard-to-reach villages.  Identify mothers and babies in families with non-smooth relationships and in dangers.  Connect those identified to healthcare service. | Approve and promote the importance of essential health care.  Make PLA discussion to prepare for support when needed. | Facilitate finance, transport, and family care coordinating local collective efforts.  Continuously monitor their support provision | Make home visits to difficult families and provide advice and emotional support. |
| *Note: HEWs = Health extension workers, HDTs = Health development team, COLs = Community opinion leaders (religious, cultural, local welfare scheme, and administrative leaders working as a group to ensure support mechanisms), PLA = Participatory Learning and Action (a community problem solving model)* | | | | |

**Outcome and process indicators**

The primary maternal outcome measure is the completion rate of essential maternal care. We define a continuum of care completion as follows:

1. fully completed care when a mother has received four or more antenatal care, delivered in a health facility with a skilled attendant, and received at least four postnatal care (first 24 hours, 48-72 hours, 7-14 days, and six weeks after delivery) from HEWs or other health professionals within the first month after delivery. Because maternal completion of these essential care components will have an implication for newborn health and survival, and essential newborn care is given simultaneously during delivery and postnatal care, the completion of essential newborn care will be analyzed in relation to the maternal completion rate.
2. Emergency home-based care when the delivery occurred at home and mother and baby received immediate visit at home by HEWs or a higher health professional.
3. Sub-analysis on the primary outcome will compare intervention with control clusters separately on antenatal care completion rate, rate of facility delivery, proportion of assistance received immediately form HEWs or other professionals when a birth occurred at home, and proportion of mothers who stayed at maternal waiting shelters immediate days before delivery.
4. Proportion received financial, transport, and family care support from community resource persons (COLs) when a support is critically needed.
5. The proportion of newborn babies received the four essential newborn care services (immediate and thorough drying, skin-to-skin contact, immediate breastfeeding initiation, and delayed cord clamping)
6. Emergency newborn sickness identification and referral rates.
7. Secondary outcome measure will be on whether the intervention affected reduction of neonatal mortality and stillbirth rates.

For newborn babies, we will measure and compare with the control arm the proportion of babies immediately put to breastfeeding and exclusively feeding until at least a month after birth, the proportion of babies received essential thermal care (wrapping in close, skin-to-skin contact to mother immediately, delayed baby bathing), baby resuscitation at home and in clinics, and proportion of babies received emergency treatment and referrals when they had danger signs.

**Data collection**

Data will be collected by trained independent data collectors a maximum of five times at households and from health facility records.

First, we will collect data when the mother’s gestation is 20th to 26th week when she formally recruited for subsequent follow-up. This period matches with the time when a mother became pregnant after the start of the intervention process in the intervention cluster. In other words, the mothers who enter the first data collection and follow-up phase on their 20th to 26th week of gestation has been exposed to interventions since they were pregnant. In this first round of data collection, data will be collected on basic socio-demographic and important variables for important covariates such as wealth status indicators, distance to health facility, history of healthcare utilization during the previous pregnancy and delivery.

Follow-up data collection will include the second to the fifth rounds of data collection. The second round will be 10 weeks after the first data collection (30^th^ to 36^th^ week of gestation). This data collection will mainly focus on information about variables related to the continuation of antenatal care utilization and whether the mother had pregnancy related complications and severe sickness, including information on received healthcare for sickness.

The third round of data collection is on the fourth day after delivery. The main variables in this round of data collection include variables on antenatal care completion, delivery information such as place of delivery, delivery attendant, utilization of postnatal care during the WHO recommended first 24 hours and 48-72 hours after birth, whether the mother received any financial, transport, or family care support from the COLs support system to reach to health facility, whether the mother stayed in the maternal waiting shelter nearby health facilities the immediate days before delivery, delivery outcome (livebirth or stillbirth). Additional information will include about maternal and newborn complications during delivery, essential newborn care provided during delivery (resuscitation, umbilical care, thermal care, initiation of breastfeeding, treatment for severely sick and premature babies).

The fourth round of data will be collected two weeks after delivery (10 days after the third data collection) on key first week survival, first week occurrence of morbidity, emergency help received for those who had serious sickness and utilization of postnatal care during the WHO recommended 7-14 days after birth, and essential baby care within the first two week. The final and fifth round of data collection will take place six weeks after delivery. This data collection will include variables on the first month and six week essential postnatal maternal and neonatal care, first month and six-week survival of the newborn, and information on sickness and emergency healthcare received for sickness in this period.

**Data storage and security plan**

Data will be transferred from koboCollect, a tablet-based application, directly to the data server at Hawassa University research center. Only people involved in the project will have a password-based access. Data will be de-identified of personal identifiers when stored. Personal variables will never be published anywhere. However, deidentified data will be available to researchers when requested with clear plan for use as stated in the protocol registration.

**Data entry and analysis plan**

Data will be double entered into excel sheet and transferred to R software version 4.4.1for analysis (27). Data will be analyzed and presented in a format comparing outcomes between intervention and control arms using intention-to-treat analysis, meaning all recruited will be analyzed irrespective of finishing the study process. Data analysis will use techniques that consider the clustered nature of the data.

**Dissemination plan**

We will communicate the result with local government structures, Sidama Regional State Health Bureau and publish scientific articles in internationally recognized peer-reviewed journals.

**Funding source**

Southern Ethiopia Network of Universities for Public Health (SENUPH-II) funds the study through the financial support from Norwegian Programme for Capacity Development in Higher Education and Research for Development (NORHED-II)-Project number 59360. The funders have no role in the study design, data collection, data management and analysis and interpretation of data, writing of the report, and decision to submit for publication.

**Potential strengths and limitations**

The primary strength of this trial lies in its innovative approach that implement comprehensive strategies involving a wide range of key community stakeholders. Ethiopian government system has a strong local structure with important stakeholder to support health interventions. These stakeholders include religious, cultural, administrative, and local welfare scheme leaders, who lead and influence opinions and collective resources in rural communities. They will work collaboratively alongside the community health workers and women's support groups. To improve initiation and continuum in the care, this trial proposes an integrative approach aimed at actively identifying and connecting women and newborns to routine and emergency healthcare services, with increased emphasis on women and newborns in vulnerable households and hard-to-reach villages.

The strategy focuses on strengthening existing local mechanisms to ensure initiation and continuity of essential care. This includes facilitating access to finance, transport, and family care, in addition to emotional support thereby addressing critical barriers to healthcare access.

Despite the strengths described above, the trial may face limitations due to its focus primarily on the quantitative improvement of healthcare utilization, without addressing challenges related to the quality of healthcare. The quality of healthcare measured through quality of provision and the personal experiences of service recipients are crucial factors influencing the continued utilization of healthcare services. However, given the limited scope and resources, the current trial prioritizes the initial step of connecting individuals to healthcare which in turn has the potential to create pressure demanding quality of care. We believe that creating demand through such as out trials may lead to interventions specifically targeting the improvement of healthcare quality.

# Reference

1. **References**

1. WHO. Trends in maternal mortality 2000 to 2020: estimates by WHO, UNICEF, UNFPA, World Bank Group and UNDESA/Population Division. Report February, 2023. Accessed April 3, 2024

<https://iris.who.int/bitstream/handle/10665/366225/9789240068759-eng.pdf?sequence=1>. 2023.

2. Hug L, You D, Blencowe H, Mishra A, Wang Z, Fix MJ, et al. Global, regional, and national estimates and trends in stillbirths from 2000 to 2019: a systematic assessment. The Lancet. 2021;398(10302):772-85.

3. Sharrow D, Hug L, You D, Alkema L, Black R, Cousens S, et al. Global, regional, and national trends in under-5 mortality between 1990 and 2019 with scenario-based projections until 2030: a systematic analysis by the UN Inter-agency Group for Child Mortality Estimation. The Lancet Global Health. 2022;10(2):e195-e206.

4. WHO. Levels and trends in child mortality: report 2021: estimates developed by the UN Inter-agency Group for Child Mortality Estimation. December 12, 2021. Accessed April 3, 2024. <https://www.who.int/publications/m/item/levels-and-trends-in-child-mortality-report-2021>. 2021.

5. Hug L, Mishra A, Lee S, You D, Moran A, Strong KL, et al. A neglected tragedy the global burden of stillbirths: report of the UN inter-agency group for child mortality estimation, 2020. United Nations Children’s Fund; 2020.

6. Middleton PF. Donor aid and research funding for newborn babies and preventing stillbirths. The Lancet Global Health. 2023;11(11):e1678-e9.

7. Bhutta ZA, Das JK, Bahl R, Lawn JE, Salam RA, Paul VK, et al. Can available interventions end preventable deaths in mothers, newborn babies, and stillbirths, and at what cost? Lancet. 2014;384(9940):347-70.

8. WHO. World Health Organization. Regional Office for Europe. (‎1978)‎. Declaration of Alma-Ata. World Health Organization. Regional Office for Europe. <https://iris.who.int/handle/10665/347879>. WHO Europe; 1978.

9. FMOH-Ethiopia. Health Sector Strategic Plan (HSDP-III) 2005/6-2009/10 FMOH; 2005 [Accessed April 3, 2024]. Available from: <http://www.nationalplanningcycles.org/sites/default/files/planning_cycle_repository/ethiopia/ethiopia-health-sector-development-planhsdp-iii.pdf>. Addis Ababa, 2005.

10. Assefa Y, Gelaw YA, Hill PS, Taye BW, Van Damme W. Community health extension program of Ethiopia, 2003–2018: successes and challenges toward universal coverage for primary healthcare services. Globalization and health. 2019;15:1-11.

11. Birhanu Z, Godesso A, Kebede Y, Gerbaba M. Mothers’ experiences and satisfactions with health extension program in Jimma zone, Ethiopia: a cross sectional study. BMC health services research. 2013;13:1-10.

12. Tiruneh GT, Demissie M, Worku A, Berhane Y. Predictors of maternal and newborn health service utilization across the continuum of care in Ethiopia: A multilevel analysis. PloS one. 2022;17(2):e0264612.

13. Organization WH. WHO recommendations on maternal and newborn care for a positive postnatal experience: World Health Organization; 2022.

14. Lassi ZS, Bhutta ZA. Community‐based intervention packages for reducing maternal and neonatal morbidity and mortality and improving neonatal outcomes. Cochrane database of systematic reviews. 2015(3).

15. Bhutta ZA, Soofi S, Cousens S, Mohammad S, Memon ZA, Ali I, et al. Improvement of perinatal and newborn care in rural Pakistan through community-based strategies: a cluster-randomised effectiveness trial. The Lancet. 2011;377(9763):403-12.

16. Midhet F, Becker S. Impact of community-based interventions on maternal and neonatal health indicators: Results from a community randomized trial in rural Balochistan, Pakistan. Reproductive health. 2010;7:1-10.

17. Mushi D, Mpembeni R, Jahn A. Effectiveness of community based safe motherhood promoters in improving the utilization of obstetric care. The case of Mtwara Rural District in Tanzania. BMC pregnancy and childbirth. 2010;10:1-9.

18. Kerber KJ, de Graft-Johnson JE, Bhutta ZA, Okong P, Starrs A, Lawn JE. Continuum of care for maternal, newborn, and child health: from slogan to service delivery. The Lancet. 2007;370(9595):1358-69.

19. Areru HA, Dangisso MH, Lindtjørn B. Births and deaths in Sidama in southern Ethiopia: findings from the 2018 Dale-Wonsho Health and Demographic Surveillance System (HDSS). Global Health Action. 2020;13(1):1833511.

20. UNdata. Statistics: Crude birth rate (births per 1,000 population), Ethiopia, 2024. Available at: <https://data.un.org/Data.aspx?d=PopDiv&f=variableID%3A53>. Accessed 08 July 2024. UNdata. 2024.

21. Cazottes I, Costello A, Davis J, George A, Houeto D, Howard-Grabman L, et al. WHO recommendation on community mobilization through facilitated participatory learning and action cycles with women s groups for maternal and newborn health. World Health Organization; 2014.

22. Pulkki-Brännström A-M, Haghparast-Bidgoli H, Batura N, Colbourn T, Azad K, Banda F, et al. Participatory learning and action cycles with women’s groups to prevent neonatal death in low-resource settings: A multi-country comparison of cost-effectiveness and affordability. Health Policy and Planning. 2020;35(10):1280-9.

23. Breuer E, Lee L, De Silva M, Lund C. Using theory of change to design and evaluate public health interventions: a systematic review. Implementation Science. 2015;11:1-17.

24. Diego EK, Ehret DE, KC A, Bose CL. Quality Indicators to Evaluate Essential Newborn Care in Low-and Middle-Income Countries. Pediatrics. 2023;152(3).

25. Wojcieszek AM, Bonet M, Portela A, Althabe F, Bahl R, Chowdhary N, et al. WHO recommendations on maternal and newborn care for a positive postnatal experience: strengthening the maternal and newborn care continuum. BMJ Global Health. 2023;8(Suppl 2):e010992.

26. Hemming K, Girling AJ, Sitch AJ, Marsh J, Lilford RJ. Sample size calculations for cluster randomised controlled trials with a fixed number of clusters. BMC medical research methodology. 2011;11:1-11.

27. R Core Team (2021). R: A language and environment for statistical computing. R Foundation for Statistical Computing, Vienna, Austria. Available at: <https://www.R-project.org/>.

28. Abrahim O, L.E., Mohammed H, Fetene N, Bradley E, *A Patient-Centered Understanding of the Referral System in Ethiopian Primary Health Care Units.* 2015.

29. Health, F.M.o., *National Referral System Network Development Liaison officer reference manual* 2015.

30. Mesganaw Fantahun Afework, K.A., Alemayehu Mekonnen, Seifu Hagos, Meselech Asegid and Saifuddin Ahmed, *Effect of an innovative community based health program on maternal health service utilization in north and south central Ethiopia: a community based cross sectional study.* 2014.

31. January, F.D.R.o.E.M.o.H., *BEmONC -Training manual JUNE 8 2018.* 2018.

32. Peter Waiswa1, 3, Karin Kallander1,2, Stefan Peterson1,2,4, Goran Tomson1,5 and George W. Pariyo2, *Using the three delays model to understand why newborn babies die in eastern.* 2010.

33. Hayelom Gebrekirstos Mengesha1, a.B.W.S., *Cause of neonatal deaths in Northern Ethiopia: a prospective cohort study.* 2017.

34. Hagos Tasew1*, M.Z., Girmay Teklay1, Teklewoini Mariye1 and Ebud Ayele1, *Risk factors of birth asphyxia among newborns in public hospitals of Central Zone, Tigray, Ethiopia 2018.* 2018.

35. Sumiyo Okawa, 2 Hla Hla Win,3 Hannah H Leslie,4 Keiko Nanishi,5 Akira Shibanuma,1 Phyu Phyu Aye,6 Masamine Jimba1, *Quality gap in maternal and newborn healthcare: a cross-sectional study in Myanmar.* BMJ Global Health 2019.

36. World Health Organization, *STANDARDS FOR IMPROVING QUALITY OF MATERNAL AND NEWBORN CARE IN HEALTH FACILITIES.* 2016: p. 14.

37. Sumiyo Okawa, 2 Margaret Gyapong,3,4 Hannah Leslie,5 Akira Shibanuma,  1 Kimiyo Kikuchi,  1,6 Francis Yeji,7 Charlotte Tawiah,8 Sheila Addei,3 Keiko Nanishi,1,9 Abraham Rexford Oduro,7 Seth Owusu-Agyei,8 Evelyn Ansah,4,10 Gloria Quansah Asare,11 Junko Yasuoka,1,12 Abraham Hodgson,10 Masamine Jimba,1 on behalf of Ghana EMBRACE Implementation Research Project Team, *Effect of continuum-of-care intervention package on improving contacts and quality of maternal and newborn healthcare in Ghana: a cluster randomised controlled trial.* 2019.

38. Bryce, E., et al., *Coverage of the WHO's four essential elements of newborn care and their association with neonatal survival in southern Nepal.* BMC Pregnancy Childbirth, 2020. **20**(1): p. 540.

39. Olaniran, A., et al., *The roles of community health workers who provide maternal and newborn health services: case studies from Africa and Asia.* BMJ Glob Health, 2019. **4**(4): p. e001388.

40. International Labour Organization, *International Standard Classification of Occupations (ISCO-08): structure, group definitions and correspondence tables. Geneva: International Labour Organization,*[*http://www.ilo.org/wcmsp5/groups/*](http://www.ilo.org/wcmsp5/groups/) *public/---dgreports/---dcomm/---publ/documents/publication/ wcms_172572.pdf (accessed July 2, 2022).* 2012.

41. Ayete-Nyampong, J. and E.A. Udofia, *Assessment of knowledge and quality of essential newborn care practices in La Dade Kotopon Municipality, Ghana.* PLoS One, 2020. **15**(8): p. e0237820.

42. Briggs, D.C., A.U. Eneh, and E.A.D. Alikor, *Basic neonatal resuscitation: retention of knowledge and skills of primary health care workers in Port Harcourt, Rivers State, Southern Nigeria.* Pan Afr Med J, 2021. **38**: p. 75.

43. Olaniyi, A., et al., *Exploring the Perception and Experience of Neonatal Resuscitation among Community Health Extension Workers in Nigeria*. 2019.

44. World Health Organization, *Community health workers: a strategy to ensure access to primary health care service.* 2016.

45. World Health Organization, *WHAT DO WE KNOW ABOUT COMMUNITY HEALTH WORKERS? A SYSTEMATIC REVIEW OF EXISTING REVIEWS* 2020.

46. Horwood, C., et al., *A continuous quality improvement intervention to improve the effectiveness of community health workers providing care to mothers and children: a cluster randomised controlled trial in South Africa.* Hum Resour Health, 2017. **15**(1): p. 39.

47. Okereke, E., et al., *Reducing maternal and newborn mortality in Nigeria-a qualitative study of stakeholders' perceptions about the performance of community health workers and the introduction of community midwifery at primary healthcare level.* Hum Resour Health, 2019. **17**(1): p. 102.

48. Namazzi, G. and M. Okuga, *Working with community health workers to improve maternal and newborn health outcomes: implementation and scale-up lessons from eastern Uganda.* 2017. **10**(sup4): p. 1345495.

49. Kok, M.C., et al., *A qualitative assessment of health extension workers' relationships with the community and health sector in Ethiopia: opportunities for enhancing maternal health performance.* Hum Resour Health, 2015. **13**: p. 80.

50. Bereket Mathewos, H.O., 2 Deborah Sitrin,3 Simon Cousens,2 Tedbabe Degefie,3 Stephen Wall,3 Abeba Bekele,1 Joy E Lawn2 and Emmanuelle Daviaud4,*, *Community-Based Interventions for Newborns in Ethiopia (COMBINE): Cost-effectiveness analysis.* 2017.

51. Ethiopia, T.G.o., *Health Policy of Ethiopia.* 1993

52. Lassi, Z.S., et al., *Interventions to Improve Neonatal Health and Later Survival: An Overview of Systematic Reviews.* EBioMedicine, 2015. **2**(8): p. 985-1000.

53. Guta, Y.R., et al., *Community-based maternal and newborn care: A concept analysis.* Curationis, 2018. **41**(1): p. e1-e6.

54. WHO, *Primary Health Care , NOW MORE THAN EVER.* 2008.

55. Wuneh, A.D., et al., *Wealth-based equity in maternal, neonatal, and child health services utilization: a cross-sectional study from Ethiopia.* Int J Equity Health, 2019. **18**(1): p. 201.

56. Health, T.F.D.R.o.E.M.o., *Ethiopia-health-system-transformation-plan EHSTP.* 2015.

57. Miller, N.P., et al., *Integrated Community Case Management of Childhood Illness in Ethiopia: Implementation Strength and Quality of Care.* The American Journal of Tropical Medicine and Hygiene, 2014. **91**(2): p. 424-434.

58. Yenealem Tadesse, M., MPH1,Yunis Mussema Abdella, MD, MPHE2, Yared Tadesse, MD3, Bereket Mathewos, MPH MSC4, Smita Kumar, MD, MPH5, Efrem Teferi, MD, MPH6, Abeba Bekele, MD, MPH7, Abebe Gebremariam Gobezayehu, MD8, Stephen Wall MD9 *INTEGRATING CHLORHEXIDINE FOR CORD CARE INTO COMMUNITY BASED NEWBORN CARE IN ETHIOPIA* 2019.

59. Degefie Hailegebriel, T., et al., *Effect on Neonatal Mortality of Newborn Infection Management at Health Posts When Referral Is Not Possible: A Cluster-Randomized Trial in Rural Ethiopia.* Glob Health Sci Pract, 2017. **5**(2): p. 202-216.

60. children, S.t., *Saving_Newborn_Lives_Nepal_-Stories_From_the_Field_2012.* 2012.

61. Wilson, A., *Interventions to reduce maternal mortality in developing countries: a systematic synthesis of evidence.* 2014.

62. Bernt Lindtjørn, Demissew Mitiku, Zillo Zidda, Yaliso Yaya, *Reducing Maternal Deaths in Ethiopia: Results of an Intervention Programme in Southwest Ethiopia.* 2017.

63. Daniel G Datiko, E.M.B., Gemeda B Birrie, Aschenak Z Kea, Rosie Steege, M Taegtmeye, Meghan Bruce Kumar, Maryse C Kok, *Community participation and maternal health service utilization:*

*lessons from the health extension programme in rural southern Ethiopia.* 2019.

64. Ian Askew, M.W., Aisha Dasgupta, Jacqueline Darroch, Ellen Smith, John Stover, Melanie Yahnerg, *Harmonizing Methods for Estimating the Impact of Contraceptive Use on Unintended Pregnancy, Abortion, and Maternal Health.* 2020.

65. Berhan Tsegaye and A.Y. Elsabet Shudura, Alemu Tamiso, *Predictors of skilled maternal health services utilizations: A case of rural women in Ethiopia.* 2021.

66. Health, F.D.R.o.E.M.o., *Health Sector Development Program IV*. 2014.

67. Ayalneh Demissie and Y.B. Alemayehu Worku, *Predictors of facility-based delivery utilization in central Ethiopia:A case-control study.* 2022.

68. Ruth Jackson, F.H.T., Hagos Godefay, Tesfay Gebregzabher Gebrehiwot, *Health extension workers' and mothers' attitudes to maternal health service utilization and acceptance in Adwa* 2016.

69. Tesfay Gebregzabher Gebrehiwot, M.S.S., Kerstin Edin, Isabel Goicolea, *The Health Extension Program and its association with change in utilization of selected maternal health service.* 2015.

70. Yibeltal Assefa, Y.A.G., Peter S. Hill, Belaynew Wassie Taye and Wim Van Damme, *Community health extension program of Ethiopia, 2003–2018: successes and challenges toward universal coverage for primary healthcare services.* 2019

71. MERQ, *National assessment of the Ethiopian Health Extension Program.* 2019.

72. WHO, *community based intervention WHO recommendation.* 2015.

73. Lynn M. Sibley, S.T., Binyam Fekadu Desta, and A.K. Aynalem Hailemichael Frew, Hajira Mohammed, Kim Ethier-Stover, Michelle Dynes, DanikaBarry,Kenneth Hepburn, AbebeGebremariam Gobezayehu,, *Improving Maternal and Newborn Health Care Delivery in Rural Amhara and Oromiya Regions of Ethiopia Through the Maternal and Newborn Health in Ethiopia Partnership.* 2015.

74. Patience A. Afulani, L.B., Francisca Essandoh,Joyceline Kinyua,Leah Kirumbi and Craig R. Cohen, *Quality of antenatal care and associated factors in a rural county in Kenya: an assessment of service provision and experience dimension* 2019.

75. Lauren Y Maldonado, et al., *Improving maternal, newborn and child health outcomes through a communitybased women’s health education program: a cluster randomised controlled trial inwestern Kenya.* 2020.

76. Erin Sines, U.S., Steve Wall, and Heidi Worley, *Postnatal Care: A Critical Opportunity to Save Mothers and Newborns.* 2007.

77. Hiwot Abera Areru, M.H.D., Bernt Lindtjørn, *Low and unequal use of outpatient health services in public primary health care facilities in southern Ethiopia: a facilitybased cross-sectional study.* 2021.

78. Aveling, E.-L., et al., *Optimising the community-based approach to healthcare improvement: Comparative case studies of the clinical community model in practice.* Social Science & Medicine, 2017. **173**: p. 96-103.

79. Agonafir, M., et al., *Community Based Essential Newborn Care Practices and Associated Factors among Women Who Gave Birth at Home in Last 12 Months in Amaro Woreda, Southern Ethiopia, 2019.* Glob Pediatr Health, 2021. **8**: p. 2333794X211016151.

80. Habte, A., K. Lukas, and T. Tamirat, *The level of Community-Based Essential Newborn Care utilization and associated factors among rural women in Southern Ethiopia, 2020: Based on the updated Community-Based Essential Newborn Care guideline.* SAGE Open Medicine, 2022. **10**: p. 205031212110676.

81. Amsalu, E.T., et al., *The effects of ANC follow up on essential newborn care practices in east Africa: a systematic review and meta-analysis.* Sci Rep, 2021. **11**(1): p. 12210.

82. Masaba, B.B. and R. Mmusi-Phetoe, *<p>Neonatal Survival in Sub-Sahara: A Review of Kenya and South Africa</p>.* Journal of Multidisciplinary Healthcare, 2020. **Volume 13**: p. 709-716.

83. Alaka Adiso Limaso1, M.H.D.a.D.T.H., *Neonatal survival and determinants of mortality in Aroresa district, Southern Ethiopia: a prospective cohort study.* 2020.

84. Lawn, J.E., S. Cousens, and J. Zupan, *4 million neonatal deaths: when? Where? Why?* Lancet, 2005. **365**(9462): p. 891-900.

85. Achamyelesh G, et al., *Health Extension Workers Involvement in the Utilization of Focused Antenatal Care Service in Rural Sidama Zone, Southern Ethiopia: A Cross-Sectional Study* Health Services Research andManagerial Epidemiology 2019. **1-8**.

# Annex I:

Table1: Estimated sample size for each objective

| Study objectives | Population and design | Assumptions | Sample size |
| --- | --- | --- | --- |
| 1. To assess the feasibility of SDG 3 in achieving a reduction of maternal and neonatal mortality by 2030 in Ethiopia | Systematic review | The target set by the SDG3 and current achievements using published articles | Not limited |
| 1. To assess baseline knowledge and skill of Health Extension Workers and Community Health workers on community-based maternal, neonate and child health service | HEWs in the selected districts, cross sectional | All HEWs in the selected districts will be eligible for this study | All HEWs  600CHWs |
| 1. To evaluate the effect of interventions package in improving the Health Extension workers satisfaction | HEWs in the selected, cross sectional. | All HEWs in the selected districts will be eligible for this study | All HEWs |
| 1. To evaluate cost and cost effectiveness of improving knowledge and skills of HEWs and community health development teams on community based maternal and neonatal health care service provision | Pregnant women and their neonate | Direct and indirect cost estimation for community based maternal and neonatal health care service quality improvement | 941 |

**Annex II List of costs**

**Table 2. Cost of material needed for intervention / cost is not yet confirmed with current price of the market.**

| **S.N** | **Material /supply** | **Quantity** | **Unit cost** | **Total** |
| --- | --- | --- | --- | --- |
| **1** | **Thermometer** | **20** | **1750** | **35000** |
| **2** | **Sphygmomanometers/anaroid** | **20** | **3699** | **73980** |
| **3** | **Fetoscop/** | **20** | **3600** | **72000** |
| **4** | **Iron folate** | **1pack/3 dose/women**  **20*3** | **39** | **2340** |
| **5** | **Baby weight scale**( 25kg Unicef Center baby weighing scale) | **20** | 3950 | **79000** |
| **6** | Ambu bag and mask | 20 | 2400 | 48.000 |
| **7** | Manual suction(bulb) | 20 ($10 for each) | 530 | 10,600 |
| **8** | Cord tie | 25*20 = 500 | 6.50 | 3.250 |
| **9** | Baby weighing bag made of cloth | 20 | 200 | 4000 |
| **10** | Glove disposable | 20* 2 box | 540 | 21600 |
| **11** | **Dipstick** | **20*2** | **340** | **13600** |
| **12** | **Albendazol** | **20*3** | **253** | **15180** |
| **13** | **Glove disposable** | **20* 2 box** | **540** | **21600** |
| **14** | **Amoxicillin syrup** | **20* 42/HF** | **28.50** | **23940** |
| **15** | **Cotrimoxazol syrup** | **20*42** | **28** | **23520** |
| **16** | **Logbook** | **20*2 one for newborn one for mother** | 300*20 | 6,000 |
|  | **Total** |  |  | **453,610** |

# Annex III: Study Information sheet

**Title**: Evaluate and determine the effect of community- based maternal and neonatal health care interventions in terms of maternal and neonatal outcomes at Sidama Regional state in 2022 -2023.A cluster randomized controlled trial. It is a **SiMaNeH-**Trial as part of SENUPH-II maternal and child health project at Hawassa University in collaboration with University of Bergen.

Greetings! My name is _________________. I am working as a data collector of PhD student, Hirut Gemeda who is learning at Hawassa University, College of Medicine and Health science, School of public health. Her supervisors are:

- Dr. Achamyelesh G/Tsadik, Hawassa University, Ethiopia
- Dr. Yaliso Yaya, Faculty of Health and Social Sciences, Western Norway University of Applied Sciences (hvl.no/en)
- Hirut Gemeda , Hawassa University, Ethiopia
- Yemisrach Shiferaw, Hawassa University, Ethiopia

**The Objective of the study:** the study will evaluate and determine the effect of community- based neonatal health care interventions in terms of neonatal outcomes at Sidama Regional, Ethiopia

**Procedure:** The study involves interviewer-administered questionnaire with the data collector that will ask you a set of questions using a structured questionnaire. After signing the consent form, the Data collector will then ask you the relevant questions and your responses will be written on the questionnaire. The interview will take about 30 minutes.

**The Risks and Benefit of the study:** There is no any risk or discomfort that you will face by participating in this research except dedication of time for responding. Any personal information registered will be not be transferred to other bodies and kept confidential. Though there is no direct benefit from this research project, the finding of this study will used to implement intervention and reveal out gap regarding community-based neonatal health care.

**Follow- up time:** The neonate will be followed until the end of 28^th^ day. During the postnatal period, newly delivered neonates will be visited five times. This schedules are: at 1st, 3rd, 7th, 14^th^ and 28^th^ day of birth.

**Privacy, anonymity and confidentiality:** Your name will be kept confidential. The study forms will be kept for five years in the project office in locked cabinet. Only research staff will be able to see those forms. Mrs. Hirut Gemeda is the principal investigator of this research.

**The Rights of participants:** completely free to take part or not in this study. If you decide that you do not want to be part of the study, you are welcome. You are also free to withdraw from the study at any time if you feel that you cannot proceed. Even if you do not want to join the study, you will receive the same quality of medical care from governmental health facilities.

**Compensation:** you will not be paid for your participation this study**.** If you agree to our proposal of enrolling your neonate in our study, please indicate that by putting your signature or your left thumbprint at the specified space below. Have you agreed to participate in the research?

1- No (say thank you) 2- Yes (take informed consent)

# Annex IV: Informed consent

The objective, benefits, harms, procedures and confidentially of the study has been read and explained to me in the language I comprehend. I further understand that, taking part in this study and withdraw from participating in any time without having reason is purely voluntary. I agree to participate in this study.

Participant:

_______________________________________________ __________________

Signature or left thumbprint of participant Date

________________________________________________ _______

Signature of the interviewer (Data collector) Date

Thank you for your cooperation

The signed copies must be given 1) to the PI and 2) to participant
