# Supplementary material for: Cluster randomized controlled trial to assess the effectiveness of a package of community-based interventions on continuum of maternal and newborn healthcare in Sidama, Ethiopia: The SiMaNeH trial protocol
Source: PLoS One. 2025 Apr 17;20(4):e0310341. doi: 10.1371/journal.pone.0310341 (PMC12005492; doi:10.1371/journal.pone.0310341)
Supplement: Attachment — Submitted filename: Point by point response for protocol manuscrpt 2.docx [file pone.0310341.s003.docx]

**Date 1/11/2024**

**Point to point Responses for reviewer comments**

**We really appreciate your constructive and insightful comments.**

| - Editor   **Comments to Author** | **Response** |
| --- | --- |
| Please amend the title either on the online submission form or in your so that they are identical. | Thank you for your comments. It is done |
| When completing the data availability statement of the submission form, you indicated that you will make your data available on acceptance. We strongly recommend all authors decide on a data sharing plan before acceptance, as the process can be lengthy and hold up publication timelines. Please note that, though access restrictions are acceptable now, your entire data will need to be made freely accessible if your manuscript is accepted for publication. This policy applies to all data except where public deposition would breach compliance with the protocol approved by your research ethics board. If you are unable to adhere to our open data policy, please kindly revise your statement to explain your reasoning and we will seek the editor's input on an exemption. Please be assured that, once you have provided your new statement, the assessment of your exemption will not hold up the peer review process. | Dear Editor,  Thank you very much for raising this important point. As this is a proposal, we do not have data at hand. However, once we collect the data and submit a manuscript for publication, we will be happy to provide it upon request. |
| Please ensure that you refer to Table 1 in your text as, if accepted, production will need this reference to link the reader to the Table. | Thank you table 1 already cited in the text. |
| The concern of the journal office is not Figure 1 as a whole, but rather the underlying map image used to construct Figure 1. Please specify the source of the map used to construct Figure 1 and its copyright status. | Thank you for your request. The authors created the map using ArcGIS software. The shape file is freely available online; however, it originates from the Central Statistical Agency, and we have cited that source in the map. |
