# Supplementary material for: Cluster randomized controlled trial to assess the effectiveness of a package of community-based interventions on continuum of maternal and newborn healthcare in Sidama, Ethiopia: The SiMaNeH trial protocol
Source: PLoS One. 2025 Apr 17;20(4):e0310341. doi: 10.1371/journal.pone.0310341 (PMC12005492; doi:10.1371/journal.pone.0310341)
Supplement: Attachment — Submitted filename: Rebutal document 3.docx [file pone.0310341.s004.docx]

**Dear Editor,**

Thank you for providing us additional comment by inviting third new reviewer and for resubmission of our manuscript PONE-D-24-34008 entitled “Cluster randomized controlled trial to assess the effectiveness of a package of community-based intervention on continuum of maternal and newborn healthcare in Sidama, Ethiopia: The SiMaNeH trial protocol.”

We appreciate the new observation of on our manuscript which makes the protocol more clear and understandable to the scientific community.

Below follows a point-by-point rebuttal to each comment from the reviewer.

**Reviewer comment**

There are couple of infelicities in the English - eg the spare word Ethiopia in the abstract (end of line 4 or the background; tense on line 3-4 of setting part of methods etc). Please carefully copyedit the manuscript

**Response**: Thank you for your comment as per your suggestion English is revised in the abstract and background section incudiding the entire document

**Reviewer comment**

I'm unclear in Table 1 how this relates to Figure 1 - is this the expected birthrate of the red zone or of the purple and yellow zones. This needs moer explanation, and the sizes of the different Kebeles given to ensure proper balance and range of sizes. The buffer zones - and the fact there are not universally buffers between Kebeles in different arms needs to be explained as well as Figure 1 is introduced.
**Response**

Thank you for your insightful observations. We appreciate your concerns and would like to provide further clarification regarding Table 1 and Figure 1.

Firstly, Table 1 presents the expected number of pregnant mothers we anticipate evaluating from a specific kebele within a designated time period. This data is crucial for assessing our intervention outcomes. In contrast, Figure 1 illustrates the protection against information contamination by creating buffer zones, which was developed using ArcGIS.

Regarding the size differences among kebeles, our goal is to balance the total population between the intervention and control kebeles. It is important to note that achieving equal sizes for each kebele is not feasible, as two kebeles will inherently differ in size.

We acknowledge the need to explain the buffer zones and the fact that these zones may not be universally applicable between kebeles in different arms. Lastly, we confirm that there is a clear demarcation between kebeles, with each kebele having clear borders.

**Reviewer comment**

Please justify an ICC of 0.01 - where has this been taken from, and please demonstrate it is relevant to this setting.

**Response**

Thank you for your valuable suggestion. We have included the reference in the sample size determination section to clarify the justification for using an ICC of 0.01. This reference provides context and demonstrates its relevance to our specific setting.

**Reviewer comment**

We are told that 2000 pairs will be used here, but 150 per cluster - please discuss the discrepancy here.

**Response**

Thank you for your critical observation. You are correct that the total number of pairs needed is 2,000, translating to 1,000 pairs per arm. The figure of 150 refers to the expected average number of births in our study clusters. Specifically, the calculated average number of births during the study period was estimated to be 100 per cluster. However, this number may vary, potentially being higher or lower, depending on the total population within each kebele.

**Reviewer comment**

There is an issue with individual consent and cluster randomization in terms of differential consent by arm - one generally needs to explain how this is to be guarded against and what the expected consent rates are going to be - will willingness to consent increase the ICC? Is there any way of getting some data from routine records? Completion of visits is presumably routinely reported enabling an ITT analysis.The issue of precisely what data is to be collected and how analyzed is missing.

**Response**

Thank you for highlighting this important issue. We appreciate your concerns regarding individual consent and the potential for differential consent rates by arm, which could impact outcome assessments.To address this, we have incorporated the non-response rate into our sample size calculations and have planned for an intention-to-treat (ITT) analysis. Additionally, we aim to collect facility data related to antenatal care (ANC) and the timing of discharge after delivery for mothers who give birth at health facilities and have initiated ANC.We believe these measures will help mitigate the effects of non-response and ensure robust analysis throughout the study. Thank you for your understanding.

Kind regards.
